# PReS-FINAL-2285: Pediatric non-renal SLE presenting as periorbital edema: response to treatment with belimumab

**DOI:** 10.1186/1546-0096-11-S2-P275

**Published:** 2013-12-05

**Authors:** B Arabshahi

**Affiliations:** 1 Department of Pediatrics, Section of Rheumatology, Inova Fairfax Hospital for Children, Fairfax, VA, USA

## Introduction

Periorbital edema without proteinuria is a rare and often difficult to manage manifestation of SLE. The use of belimumab for treatment of this complication has not been previously described in the pediatric literature.

## Objectives

To describe the use of belimumab for treatment of steroid-dependent chronic periorbital edema in new onset non-renal SLE.

## Methods

Case report: A 16 year old white female presented with a three month history of periorbital edema in the setting of positive lupus serologies, synovitis, and hypocomplementemia. She had failed treatment with anti-histamines and had responded well to the use of oral steroids, but her symptoms quickly recurred upon tapering off steroids. Over the following six months, treatment with hydroxychloroquine and methotrexate failed to provide any steroid-sparing benefit. Addition of a three month trial of omalizumab also failed to provide any benefit. IV belimumab at 10 mg/kg monthly, was initiated and continued upon one year follow-up.

## Results

Treatment with belimumab resulted in complete resolution of the patient's periorbital edema, allowing her to discontinue the use of steroids within a month of starting this agent. No adverse reactions have been observed upon one-year follow up, and the patient's symptoms remain well controlled off steroids.

## Conclusion

Belimumab may serve as a safe and effective steroid sparing agent for management periorbital edema associated with non-renal SLE.

## Disclosure of interest

None declared.

